# Psychological Distress and Online Academic Difficulties: Development and Validation of Scale to Measure Students’ Mental Health Problems in Online Learning

**DOI:** 10.3390/bs15010026

**Published:** 2024-12-30

**Authors:** Mihai Curelaru, Versavia Curelaru

**Affiliations:** 1 Department of Psychology, Faculty of Psychology and Educational Sciences, Alexandru Ioan Cuza University of Iasi, 700506 Iasi, Romania; 2 Department of Teacher Training, Faculty of Psychology and Educational Sciences, Alexandru Ioan Cuza University of Iasi, 700506 Iasi, Romania; versavia.curelaru@uaic.ro

**Keywords:** mental health problems, psychological distress, online academic difficulties, online learning, COVID-19, scale development

## Abstract

In the present study, a short instrument (eight-item self-report, five-point Likert scales) was developed and validated to assess self-perceived mental health problems in online learning. The participants were 398 Romanian university students from nine different faculties. The factor structure of the scale was assessed using Exploratory Factor Analysis (Principal Axis Factoring extraction method) and Confirmatory Factor Analysis. The high goodness-of-fit indices validated a second-order factor model of mental health problems, with two distinct but correlated sub-constructs, psychological distress, and online academic difficulties, integrated under a single higher-level factor. Psychological distress comprises indicators such as anxiety and stress, while online academic difficulties contain, for instance, indicators such as decreased performance, fatigue or lack of motivation. The results of applying multiple assessment criteria showed good reliability (e.g., McDonald’s omega), as well as convergent validity (e.g., Average Variance Extracted) and discriminant validity (e.g., the heterotrait–monotrait ratio of correlations) of the scale. Also, correlations analysis between mental health problems occurred in online learning context and other measures indicated a strong negative relation with online course satisfaction and weak negative relations with subjective academic performance, perceived social competence, and perceived digital competence. In conclusion, the scale appears to be a valid instrument for measuring some negative mental health outcomes in online learning, perceived by university students. The implications of the results and limitations of this study are also discussed. In conclusion, the scale has multiple possible applications, the most important being (1) the assessment of mental health problems both in ordinary online learning situations and in emergency ones, which would allow the early detection of these issues, (2) the possibility of assessing relations between the sub-constructs of the scale and other psychological constructs of interest in scientific research, and (3) the feedback for teaching staff involved in the online learning system.

## 1. Theoretical Background

The early recognition of some mental health problems (MHPs) and the factors that affect psychological and social well-being, as well as the identification of protective measures, must be a constant concern of public health institutions, but also of the educational system.

Adolescence and early adulthood are crucial for psychological and social development, but it is also a period of greater vulnerability to MHPs. Statistics from 2022 in the U.S. estimated that young adults between 18 and 25 years old have a higher prevalence of mental illness in comparison to adults over this age (National Institute of Mental Health, 2024). Worldwide, it is also estimated that 50% of mental disorders occur from the age of 14, while 75% of them occur from the age of 24 (Institutul National de Sanatate Publica, n.d.) and that 14% of 10–19-year-olds experienced mental health conditions (World Health Organization, 2024). A study conducted in England between 2017 and 2022 showed an increase in MHPs among young people aged 17 to 24 years, with 22.0% of them having a probable mental disorder and 13.6% having a possible mental health disorder (NHS England, 2022). Statistical evidence in recent years regarding the increase in MHPs has raised concerns for prevention and intervention at a university level as well, where anxiety and stress produced by academic demands, greater competition, insecurity about future careers, or economic pressures have been highlighted (Eleftheriades et al., 2020). The COVID-19 pandemic and the sudden widespread take-up of the synchronous online learning education have brought new challenges for university students and created new learning situations that need to be carefully considered in relation to mental health. For example, developing cognitive and emotional self-regulation skills should be an important educational objective for university students in the context of online learning, which requires a high level of autonomy. Studies show that skills such as planning, self-monitoring, time management, or emotional balance in situations of stress and anxiety are important for reducing the tendency to procrastinate, a phenomenon with high prevalence in students and with negative effects on academic performance and psychological well-being (Diotaiuti et al., 2021).

### 1.1. Mental Health

Mental health is a complex concept with a wide variety of definitions, theoretical models, and languages used to describe them. In this section, we will briefly present two of the most commonly used perspectives to approach this concept.

In a *narrower, negative, and exclusive perspective*, the central idea in the conceptualization of mental health is the absence of mental illness. In other words, mental health diagnosis is based on the presence of symptoms of mental illness and the negative effects of this in a person’s life. Mental health state is assessed using a number of indicators, of which *psychological distress* is a widely used one (Drapeau et al., 2012). A scoping review showed that psychological distress is one of the most commonly used indicators in the assessment of mental health, in particular through two of its core elements: anxiety and depression (Peitz et al., 2021). Other authors also support this idea, such as Tannenbaum et al. (2009), who state that depression and anxiety can be considered “as prototype mental health symptoms/disorders” (p. e179). Psychological distress is a label that covers a wide range of psychological manifestations, from the subclinical to clinical symptoms of depression, anxiety, and stress (Zhu et al., 2022). Defined as “a set of painful mental and physical symptoms that are associated with normal fluctuations of mood in most people” (American Psychological Association, 2018) or as “a range of symptoms and experiences of a person’s internal life that are commonly held to be troubling, confusing, or out of the ordinary” (American Psychiatric Association, 2013, DSM-5, p. 827), psychological distress has been considered both a transient phenomenon manifested by emotional turmoil due to the inability to cope with stressful situations, and a diagnostic criterion in certain psychiatric disorders (Drapeau et al., 2012). In an educational context, psychological distress has been operationalized by anxiety and depressive symptoms (Knapstad et al., 2021), depressive symptoms, anxiety, and stress (Kumar et al., 2016; Naylor, 2020) or anxiety, depression, and suicidal ideations (Schmits et al., 2021). In recent years, this perspective on psychological distress has become less restrictive, including a lack of well-being. For instance, Burnette et al. (2020) define psychological distress as “symptoms of anxiety, depression, psychological stress, or absence of well-being” (Burnette et al., 2020, p. 1). The new view of mental health was further developed by adding to the two core elements of psychological distress, anxiety and depression, and other indicators such as anger and social support (Young et al., 2007) and loneliness, suicidal ideation and attempts (Pengpid & Peltzer, 2020).

From a *broader, positive, and integrative perspective*, mental health was conceptualized in terms of *well-being*. In this case, mental health refers to our emotional, psychological, and social well-being, which influences how we think, feel, and behave, how we manage stress, relate to others, and make healthy choices (Centers for Disease Control and Prevention, 2024). Promoters of this mental health perspective state that for a long time, mental health has been associated with the lack of mental disorders and less with the presence of well-being as a consequence of cultivating positive emotions, character, and life satisfaction (Cloninger, 2006). This positive psychology approach to mental health is also reflected in the World Health Organization’s report (2022) which defines it as “a state of mental well-being that enables people to cope with the stresses of life, realize their abilities, learn well and work well, and contribute to their community.” (p. 8). In the same perspective, mental health was defined by a set of adaptive functions that contribute to human developmental goals and psychological well-being, such as perception, abstract thinking, emotions, and the ability to think logically, categorize objects, ascribe beliefs and desires to other people, to develop motivations to act, as well as to cooperate and use language” (Schramme, 2010, p. 39).

Within this conceptual perspective, two research directions have emerged. In one of them, mental health is seen as a bipolar construct. It is based on a single-continuum model, with mental illness (psychological distress and psychopathological symptoms) being placed on one pole and mental health (well-being) on the other. In this orientation, well-being is the opposite of ill-being, especially its two symptoms: anxiety and depression (Huppert & So, 2013; Huppert, 2014; Mirowsky & Ross, 2003). In the second research orientation, mental health is conceptualized by a dual model. The two realities, well-being and psychopathology, are placed on a dual continuum and conceptualized as separate, unique, but related constructs, both contributing to overall mental health (Greenspoon & Saklofske, 2001). Dual-factor model of mental health is considered a better alternative to the bipolar model, because on the one hand, it integrates in a more complex way the two dimensions, mental health and mental illness. On the other hand, by crossing (or overlapping) them, it creates a typology in four clusters. Using as dimensions psychopathology (PTH) and subjective well-being (SWB), Greenspoon and Saklofske (2001) identified four groups: high SWB—low PTH, low SWB—low PTH, low SWB—high PTH, high SWB—high PTH. This typology corresponds to a later one using mental health and mental illness as dimensions. The four resulting clusters in this case are flourishing (high mental health and low mental illness), languishing (low mental health and low mental illness), languishing and mental illness (low mental health and high mental illness), and flourishing and mental illness (high mental health and high mental illness) (Keyes, 2005, 2014). To these, the cited author added two more categories, moderate mental health and, respectively, moderate mental health and mental illness (for a graphical representation, see Keyes, 2014). This mental health model has been studied in both children (Greenspoon & Saklofske, 2001) and adults, including college students (Antaramian, 2015; Eklund et al., 2011; Xiao et al., 2021). Currently, there is already a scientific debate among researchers and arguments are being formulated for both lines of research conceptualizing mental health from a well-being perspective (Iasiello et al., 2020; Zhao & Tay, 2023).

To conclude, mental health can be defined in several ways and it is no longer just an individual issue for those with a psychiatric diagnosis, but a concern for society as a whole. Everyone may experience variations in mental health across the lifespan, depending on the interaction of individual, family, community, and societal factors. We should not wait for more serious problems such as depression and anxiety, but it is important to identify as early as possible the most common signs that could indicate poor mental health. Low levels of engagement, decreases in motivation, and withdrawal from social interactions in the community, at work, or in education may be indicators of mental health deterioration for example.

### 1.2. Online Learning and Mental Health

Starting with 2019, the COVID-19 pandemic has generated a worldwide health crisis, with countries’ governments being forced to declare a state of emergency and take exceptional measures to prevent the spread of the virus. Most institutions, including schools and universities, were closed and people were isolated in their homes, online learning being a solution for continuing education in this time of crisis. The sudden shift from face-to-face to virtual education and resulting lifestyle changes (e.g., physical isolation, screen-mediated communication, learning and working at home, changes in outdoor activities, etc.) raise concerns about the structural changes in students’ behaviors and their long-term effects (De Haas et al., 2020).

The MHPs reported by the numerous studies examining student populations during the COVID-19 crisis have been related to the threat of virus infection and quarantine, but also to many other variables, including those specific to online learning: social isolation, virtual communication, prolonged exposure to screens, poor digital skills or inadequate technical support. Research carried out even before the pandemic period showed that MHPs in adolescents are associated with increasing screen time and pressure within contemporary school settings (Bor et al., 2014). Studies in recent years have highlighted many advantages of virtual education, such as flexibility in time management (e.g., Bates & Khasawneh, 2007; Song et al., 2004), increased motivation, active participation and engagement (e.g., Bates & Khasawneh, 2007; Song et al., 2004; Dumford & Miller, 2018), lower financial costs (e.g., Alexander et al., 2012; Naseer & Perveen, 2023), rapid exchanges of information (e.g., Naseer & Perveen, 2023), flexibility in task execution, accessibility and pedagogical innovation (e.g., Lucas & Vicente, 2023), etc., with this mode of teaching and learning proving its usefulness in the pandemic period when lockdown was imposed (Aisha & Ratra, 2022; Beserra et al., 2022; Mishra et al., 2020). However, a number of disadvantages of both full-online and blended systems are also mentioned in the literature, some of these referring to negative effects on learners’ mental states (Huang et al., 2024; Wang et al., 2020). A systematic review of meta-studies analyzing COVID-19-related educational experiences in the period 2020–2022 showed that, among the multiple topics of interest, student well-being was an important aspect that researchers focused on (Daumiller et al., 2023). The results highlighted an increased prevalence of anxiety, depression, and stress, as well as loneliness, alcohol use, and attention issues compared to the pre-pandemic period. The studies on college and university students showed an increase in anxiety and depression (Buizza et al., 2022; Lee et al., 2021; Zhou et al., 2021; Alam et al., 2021; Almeda et al., 2022; Wang et al., 2022; Xu & Wang, 2023), stress symptoms (Hasan & Bao, 2020; Ahorsu et al., 2020; Alam et al., 2021; Almeda et al., 2022; Xu & Wang, 2023), loneliness (Lee et al., 2021; Alam et al., 2021; Almeda et al., 2022; Pai & Vella, 2021), suicidal thoughts (Wathelet et al., 2022) or substance use (Chaffee et al., 2024). Also, low motivation was associated with online learning in the pandemic period (Soria et al., 2020) as well as poor performance of learners (Liu et al., 2022). Another research that analyzed the association of online learning with negative effects on students’ psychological state identified motivation, personal connectedness, and performance as relevant factors for mental health (Garcia et al., 2021). In a study on 209 undergraduate and postgraduate students who attended two semesters of predominantly online learning during the COVID-19 pandemic a qualitative analysis identified, in addition to self-reported symptoms of stress and anxiety, states of apathy and boredom, lower academic efficiency, tiredness and exhaustion, loneliness, as well as a decrease in motivation and ability to concentrate (Curelaru et al., 2022). States of apathy and lack of motivation could be the result of self-regulatory problems exacerbated by the blurring of external regulatory factors in the online learning environment. Or external regulatory actions (e.g., teacher’s or peers’ support) are important for promoting students’ internal self-regulation (de la Fuente et al., 2020). Self-regulation failures can affect students’ academic adaptation through the procrastination mechanism, associated with low emotional balance, low confidence and self-esteem, and high self-handicapping behaviors (Diotaiuti et al., 2021). Maloney et al. (2023) define and conceptualize online engagement fatigue among university students and teaching staff, a phenomenon that appeared in the context of the COVID-19 pandemic, characterized by general fatigue, cognitive/mental fatigue, emotional exhaustion, and burnout.

## 2. The Current Study

The main objective of this research was to develop a scale that could capture students’ perceived MHPs associated with online learning. Before presenting the concrete steps followed in the construction and validation of the scale, two conceptual clarifications are necessary. The first refers to the MHPs perspective assumed in this study, and the second to the distinction between emergency remote learning (ERL) and online learning. On the first point, it can be said from the start that, although more specific than mental health, MHPs remain a complex, broadly defined concept (Smith & McLellan, 2023). This construct is used to label a set of indicators by which mental health can be assessed. The scope of MHPs is sometimes narrower and more clearly delineated, referring to a clinical perspective, which includes psychological disorders measured with appropriate tools for their diagnosis. But the MHPs may also have a broader, non-clinical scope, including, for instance, stress or lack of well-being as indicators. Our approach is part of this second perspective. Thus, we aimed to select appropriate items for several indicators of students’ mental health involved in online learning, to test the psychometric properties of the scale, and to make suggestions for future research. On the second point, we distinguish between online learning and emergency remote learning (ERL). In online learning, three teaching modalities can be used: asynchronous online courses, synchronous online courses, and hybrid (or blended) courses. In ordinary life circumstances, these forms of course delivery are used as parts of a balanced, time-tested online instructional design that does not pose major adaptation problems for learners. But in crisis situations caused by, for example, natural disasters, pandemics, or wars, education can be delivered in a very different way. In these pressing life circumstances, ERL is the solution that allows us to continue the learning process even when both teachers and students cannot physically meet each other. The fundamental characteristics of ERL are duration limited to crisis and the fully online delivery of all academic activities. In this case, synchronous online courses are intensively used, without excluding asynchronous modality. Only blended courses are excluded from ERL because it is not possible to alternate synchronous online and in-person teaching activities. In the present study, we used the concept of online learning and not synchronous online learning or ERL because mental problems can occur not only in these situations but also in blended learning when the synchronous modality is used intensively. The mental problems presented above have been associated with online learning in the lockdown period but also in the immediate post-pandemic period, when hybrid mode education continued in many universities. The common characteristic of these two periods is the intensive use of synchronous modality. The scale we present below was created to assess MHPs in online learning not only in crisis situations or their immediate aftermath but in any other situation where signs of minimal deterioration of mental health in the broader sense of the above-mentioned term may be suspected.

Regarding the scale construction, item generation was based on two sources. First, in a theoretical/deductive manner, we formulated certain items considering the current conceptualization of mental health in the literature. Thus, in the proposed scale there are aspects related to the ability to meet daily challenges, to cope with stress, and to learn well (World Health Organization, 2022; Schramme, 2010). Then, from an empirical/inductive perspective, we developed items using the results of previous studies conducted in the pandemic and post-pandemic period, which highlighted students’ MHPs, such as anxiety, stress and loneliness (Daumiller et al., 2023; Lee et al., 2021; Almeda et al., 2022; Pai & Vella, 2021), engagement fatigue (Maloney et al., 2023), tiredness, lower academic efficiency, decrease in ability to concentrate and decrease in motivation (Curelaru et al., 2022).

The initial form of the instrument included 18 statements, with the aim of capturing student mental health issues as diversely and comprehensively as possible. To improve content validity, we sought the opinions of four education experts in order to assess, using a binary choice (yes/no), whether these items can be part of the set of mental health problems that might occur in intensive online learning. After the evaluation of these experts, nine statements were eliminated, because they were either considered to measure the lack of satisfaction of students with the courses and not the negative effects of online learning on mental health, or they were redundant, ambiguous, or irrelevant. For example, the item “Using the online learning system creates health problems for me” was eliminated because it was considered ambiguous, with the risk that the term “health” could mean physical health for some participants, mental health for others, and both for others. The nine final indicators included in the tested scale were the following: anxiety, perceived stress, insecurity, worry, difficulty concentrating, tiredness, social isolation, poor performance, and lack of motivation. We have chosen these indicators because they are relevant and specific to the online learning context. In developing the scale, we selected indicators that have emerged from empirical research rather than those that might result from adopting a theoretical model of mental health.

Consistent with the theoretical background, we decided to test, first, a structural model with two distinct, but correlated factors. We hypothesized that some indicators, such as social isolation, poor performance, and lack of motivation would aggregate into one factor, *online academic difficulties*, that could represent (lack of) well-being related to learning, and other indicators, like anxiety and perceived stress that could cluster into a second factor, *psychological distress*. Although in our theoretical perspective, we do not anticipate the existence of a one-factor model, we found it necessary to explore this possibility as well. In this second operationalization, all indicators were related directly to a central construct, *online learning mental health problems* (OLMHPs). Exploratory Factor Analysis (EFA) and then Confirmatory Factor Analysis (CFA) were performed, in order to verify the structures of these two models. We also note that in the process of validating the scale, we used another construct, online course satisfaction, to establish convergent validity. In addition to the primary objective of validating the OLMHPs scale, a secondary objective was to examine the relations between MHPs and other three variables, respectively, perceived digital competence, academic self-evaluation, and perceived social competence. We hypothesized moderate and negative associations between these variables and the OLMHPs, results that may imply some recommendations regarding the delivery of online classes.

## 3. Method

### 3.1. Participants

In this study, we used a convenient sample consisting of 398 undergraduate and postgraduate students (86.7% female), aged 20 to 60 years (M *=* 22.3, SD = 3.5), from nine different faculties of one of the largest Romanian higher education institution, Alexandru Ioan Cuza University of Iasi. Participants attended between 1 and 4 semesters of online learning (fully and blended), most of them (79.9%) attending 4 semesters (Table 1).

### 3.2. Measures

#### 3.2.1. Online Learning MHPs (OLMHPs)

A 9-item scale was built to measure the perceived MHPs that students associate with online learning. The process of developing this scale has been described in Section 2, starting with the generation of the items and ending with the elaboration of the first form of the scale that was given to the participants in this research. The wording of the scale items is presented in Section 4. Participants’ responses were collected using a five-point Likert scale (1—*strongly disagree*, 5—*strongly agree*).

#### 3.2.2. Single-Item Measures

Four other constructs (online course satisfaction, subjective academic performance, perceived social competence, and perceived digital competence) were measured by single-item scales. This kind of measure is a technique used in the global assessment of unidimensional constructs such as perceived performance, success, health status, quality of life (Bowling, 2005), personal/family life satisfaction, job satisfaction (Fisher et al., 2016), subjective academic performance, subjective socioeconomic status (Leung & Xu, 2013) or global self-esteem (Leung & Xu, 2013; Robins et al., 2001). Although measured by a single item, the data obtained can be considered reliable and valid, as shown by the results of the studies cited above. Single item technique presents several advantages, among these the most important being simplicity (Bowling, 2005), utility, efficiency, parsimonious in terms of administration time, more satisfaction for test-takers, reduction in data processing costs, reduction in ambiguity in measurement (Allen et al., 2022), minimizing respondent burden, and increasing face validity (Fisher et al., 2016). The first construct, *perceived social competence* (PSC), was defined as a perception of an individual’s own abilities to manage intrapersonal and interpersonal social and emotional experiences in order to be effective in his social interactions, to create and maintain positive social interactions with others and to achieve personal goals in these interactions (Collie, 2020; Rubin & Rose-Krasnor, 1992). This measure was a single-item scale, ranging from 1 (*very low*) to 10 (*very high*). The item was worded as follows: “Please choose a number, from 1 to 10, by which to rate your social competence (e.g., communication skills, empathy, conflict resolution, initiating new social relationships, listening to others, etc.)”. The second construct, *perceived digital competence* (PDC), represents the „confident, critical and responsible use of, and engagement with, digital technologies for learning, at work, and for participation in society” (European Commission, 2019). A student’s PDC reflects his/her perception of the ability to use Information and Communication Technologies (ICT) (e.g., communication technologies, computers, cell phones, the internet, various software applications) in the context of learning. This measure is a single-item scale, ranging from 1 (*not at all good*) to 10 (*very good*). The wording of the item was: “Please choose a number, from 1 to 10, by which to rate your digital skills (e.g., use of electronic devices such as computer, tablet or phone; use of programs installed on these devices; communication; use of the internet, etc.)”. The third measure, *subjective academic performance* (SAP), or subjective academic success, reflects the student self-assessment of their own performance. In this study, we used a 10-point single-item scale, ranging from 1 (school failure) to 10 (school success) to measure SAP, a construct whose measurement has been shown to be valid in other studies in educational settings (e.g., Leung & Xu, 2013). The item was worded as follows: “If you were to rate your performance as a student by ticking one of the numbers below, where would you place yourself: closer to academic failure (*towards 1*) or closer to academic success (*towards 10*)?”. To assess the fourth construct, *online learning satisfaction* (OLS), we also used a single-item scale, ranging from 1 (*strongly disagree*) to 5 (*strongly agree*). Learning Satisfaction (LS) represents a learner’s perceived level of fulfillment of their expectations and needs as outcomes of the learning process. The OLS construct represents a contextualization of LS in online learning, and it was measured by various multi-item scales in the pandemic and post-pandemic period (e.g., Bayrak et al., 2020; Cofini et al., 2022; Elshami et al., 2021). In our study, OLS was assessed with a single item: “In general, I am satisfied with the way the learning activities are carried out in the online system.”

### 3.3. Procedure

This research was approved by the ethics committee of the Faculty of Psychology and Educational Sciences from Alexandru Ioan Cuza University of Iasi (No. 1011/2022). Participants were given informed consent about the research objectives, data confidentiality, how to complete the questionnaire, and that they could withdraw at any time. All students who were contacted gave their consent to participate in the study. The research was conducted online between April 2022 and March 2023 using the Google Forms digital platform.

### 3.4. Statistical Analysis

Since the normality of data is an important assumption for structural equation modeling (SEM), we first evaluated the distribution of data. For this purpose, we referred to maximum values of 2 for skewness and 7 for kurtosis, recommended in the case of a sample size larger than 300 (Kim, 2013). Then, the Kaiser–Meyer–Olkin test (KMO) and Bartlett’s test of sphericity were calculated to assess the sample adequacy for factor analysis. In the next step, an Exploratory Factor Analysis (EFA) was performed to determine the factorial structure.

To validate the best-fitting model, a confirmatory factor analysis (CFA) was conducted. The values of goodness-of-fit (GOF) indices that we have used in the model verification are as follows: Relative chi-square test ≤ 3 (Kim et al., 2015; Schreiber et al., 2006), Normed Fit Index (NFI) ≥ 0.95 (Hu & Bentler, 1999), Comparative Fit Index (CFI) ≥ 0.95 (Hu & Bentler, 1999; Schreiber et al., 2006), Tucker–Lewis Index (TLI) ≥ 0.95 (Hu & Bentler, 1999; Schreiber et al., 2006), Adjusted Goodness-of-Fit Index (AGFI) ≥ 0.90 (Hooper et al., 2008), Standardized Root Mean Square Residual (SRMR) ≤ 0.05 (Byrne, 2016; Gunzler et al., 2021), and Root Mean Square Error of Approximation (RMSEA) ≤ 0.07 (Steiger, 2007).

Cronbach’s alpha (Nunnally & Bernstein, 1994) and McDonald’s omega (McDonald, 1999) were used to assess the reliability of the OLMHPs scale. In terms of reliability, McDonald’s omega is considered by some researchers as an alternative method to assess it (Hayes & Coutts, 2020), hence we decided to calculate both coefficients. The convergent validity was assessed using Average Variance Extracted (AVE) (Hair et al., 2019) and the bivariate Pearson’s correlation coefficient (r). The heterotrait–monotrait ratio of correlations (HTMT) (Henseler et al., 2015), Maximum Shared Square Variance (MSV), and Average Shared Square Variance (ASV) (Fornell & Larcker, 1981; Hair et al., 2019) were used to assess discriminant validity. In order to establish convergent and discriminant validity, we used multiple criteria, as suggested by Cheung et al. (2024).

### 3.5. Descriptive Statistics

Examination of data distribution showed that the values for skewness ranged between −0.12 and 0.82, and for kurtosis between −1.37 and −0.57, which means that there are no significant deviations from normality (Kim, 2013). For all items, the minimum and maximum response values ranged from 1 to 5, with the mean ranging from 2.22 to 3.12 (Table 2). The inter-item correlation coefficients ranged from 0.32 to 0.75, all being significant at *p* < 0.001.

## 4. Results

### 4.1. Exploratory Factor Analysis

The Principal Axis Factoring (PAF) extraction method with Varimax Rotation was used in EFA. The results of the analysis revealed two distinct factors with Eigenvalues over 1.00, accounting for 61.56% of the total explained variance. The Kaiser–Meyer–Olkin Measure of Sampling Adequacy and Bartlett’s Test of Sphericity values (KMO = 0.915, χ2 = 2043.74, *p* < 0.001) indicate that these data were applicable for exploratory factor analysis. Factor loadings ranged from 0.525 to 0.844, above the 0.50 threshold considered practically significant (Hair et al., 2019). Communalities ranged from 0.361 to 0.661, with item 4 having the smallest factor loading. The first factor (F1) included five items and explains 32.76% of the variance, while the second factor (F2), composed of four items, explains 28.80% of the variance. Cronbach’s alpha (F_1_ = 0.88, F_2_ = 0.84) and omega (F_1_ = 0.88, F_2_ = 0.84) coefficients demonstrated good reliability (Table 3).

According to Hair et al. (2019), communality coefficients obtained in EFA should have values greater than 0.50 for items to be retained in the analysis. In the present study, because item 4 had a factor loading below this threshold (0.361), it was removed from the database. Therefore, the CFA-tested model included eight items distributed in two factors, which also represent the final version of the OLMHPs scale (see Appendix A).

### 4.2. Confirmatory Factor Analysis

PAF analysis revealed a two-factor structure of the data, hence a possible bidimensional model. Other alternate used EFA extraction methods (e.g., ML) or PCA, in combination with other rotations (e.g., Promax, Quartimax) invariably provided a two-factor solution in the same item configuration of the factors. These results led us to decide to test first a correlated two-factor model (Model 1). However, the two factors resulting from the EFA, with 5 and 3 items, respectively, were highly intercorrelated (r = 0.66, *p* < 0.001). That was an argument to test in CFA, in addition to the previously mentioned model, another one, a second-order factor model (Model 2). This last model can relate the manifest variables to the first-order latent construct, which in turn can then be related to the second-order latent construct. Then, although the EFA results did not give us any serious reasons, we nevertheless tested a third model, the one initially hypothesized as a single factor order model (Model 3).

For models 1 and 2, CFA revealed identical GOF indices: χ^2^(p) = 32.60 (0.02), df = 19, χ^2^/df = 1.71, AGFI = 0.96, NFI = 0.98, TLI = 0.98, CFI = 0.99, RMSEA = 0.04, SRMR = 0.02. The same GOF indices may occur if the models had the same basic parameters, first-order factors had few variables per factor and second-order factors had few first-order factors. The two models are not discriminable in terms of their parsimony, but we could opt for one of them considering other aspects. Following several criteria, such as on the one hand the theoretical background and conceptualization, the type of structural model hypothesized, and on the other hand the EFA results, we decided to Model 2 further in the analysis. Comparing the CFA GOF indices of this model with the reference values presented in Section 3.4., we concluded that the GOF indices for construct validity achieved the required level for the retained model. Regarding Model 3, GOF indices were fallows: χ^2^(p) = 251.57 (*p* < 0.001), df = 20, χ^2^/df = 12.57, AGFI = 0.70, NFI = 0.86, TLI = 0.82, CFI = 0.87, RMSEA = 0.17, SRMR = 0.06. As can be seen, none of the GOF indices reached the minimum threshold required for model validation.

**Figure 1:**
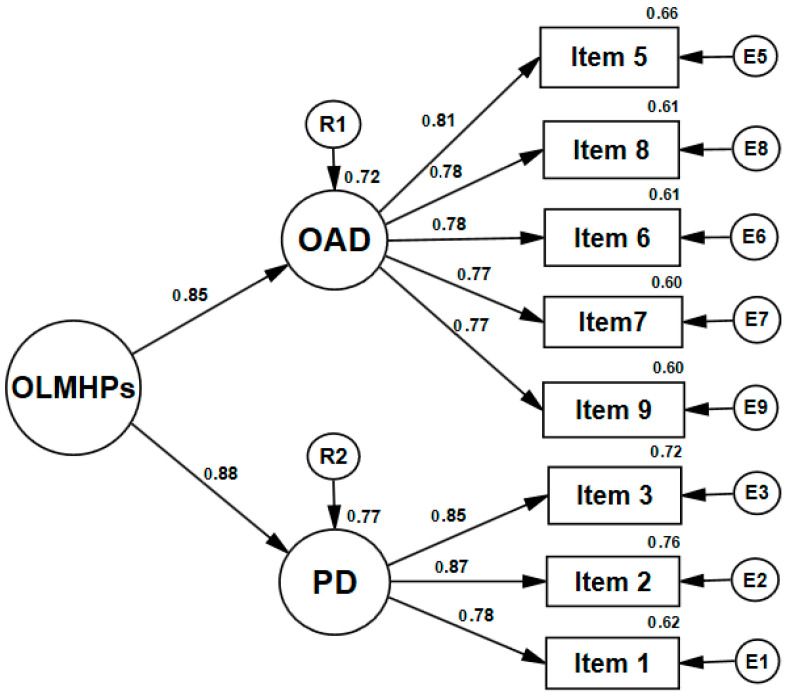
OLMHPs second-order factor model structure (Model 2).

Model 2 had two first-order factors, *online academic difficulties* (OAD) and *psychological distress* (PD), and one second-order factor, named *online learning mental health problems*. All data distributions are within normal parameters (skewness: OLMHPs = 0.22, OAD = 0.09, PD = 0.61, kurtosis: OLMHPs = −0.90, OAD = −1.05, PD = −0.64). The standardized factor loadings, ranging from 0.77 to 0.87, were all statistically significant and above the recommended minimum value of 0.50 (Hair et al., 2019). These results represent preliminary support for convergent validity. The structure of the second-order factor model appears in Figure 1.

### 4.3. Reliability

Cronbach’s alpha and McDonald’s omega coefficients were calculated to verify the reliability of the scale. Overall, the OLMHP item scale had α = 0.90 and ω = 0.91, which indicates very good reliability. Regarding the two sub-scales, α and ω were identical, 0.88 for OAD and 0.87 for PD (Table 4).

### 4.4. Convergent Validity

The factor loadings of the two OLMHPs sub-constructs had high values (λ_AD_ = 0.85, λ_PD_ = 0.88) and there was a strong correlation between them (r = 0.66, *p* < 0.001). Also, both R^2^ for OAD and PD were high (R^2^_OAD_ = 0.72 and R^2^_PD_ = 0.77), which reflects a good contribution of OLMHPs on its two sub-constructs. As regards the convergent validity of the first-order constructs, both AVE coefficients (AVE_OAD_ = 0.61, AVE_PD_ = 0.69) were above the minimum threshold of 0.50 (Fornell & Larcker, 1981) (Table 4). AVEs were larger than the squared correlation coefficient between the two sub-constructs, OAD and PD, and the relationship CR > AVE is verified for each (Table 4). Also, in terms of convergent validity, a strong relationship between two constructs is a good support for it (Fornell & Larcker, 1981; Hair et al., 2019). In our case, the bivariate Pearson’s correlation analysis showed that OLMHPs were negatively and strongly significantly associated with OLS (r = −0.62, *p* < 0.001), and similar results were obtained for its sub-constructs (r_OAD_ = −0.58, *p* < 0.001, r_PD_ = −0.57, *p* < 0.001) (Table 5). In other words, the presence of PD and OAD were equally associated with dissatisfaction with online classes. These correlations represented other results that support a good convergent validity of this scale.

### 4.5. Discriminant Validity

The discriminant validity of the OLMHPs scale was assessed using multiple criteria. First, the correlation between the two subscales (r = 0.66, *p* < 0.001) was less than 0.80, which indicates a good discriminant validity (Brown, 2015). The second way to verify discriminant validity was to examine Model 3, with a single factor, in which the two dimensions are collapsed into a one-dimensional model. It has already been noted that the GOF indices do not pass the minimum accepted thresholds. Therefore, a first-factor model is invalidated and two distinct dimensions must be accepted. Third, the 0.75 value of the HTMT coefficient between the two reflective sub-constructs was lower than the 0.90 threshold suggested in the literature (Hair et al., 2019), which shows a good discriminant validity of the scale. The last way to verify the discriminant validity was to assess the Maximum shared squared variance (MSV), the Average shared squared variance (ASV), and the relationship between them and AVE. The shared variance within constructs should be greater than the shared variance between constructs (Fornell & Larcker, 1981; Hair et al., 2019). In our study, MSV and ASV were both 0.44, therefore lower than AVEs, whose coefficients were 0.61 and 0.69. These results provide evidence for good discriminant validity between the two sub-constructs.

### 4.6. Correlations with Other Measures

As seen in Table 5, the bivariate Pearson’s correlation analysis showed that OLMHPs were negatively and significantly associated with PSC (r = −0.18, *p* < 0.001), PDC (r = −0.27, *p* < 0.001), and SAP (r = −0.24, *p* < 0.001). In addition, the correlations between OAD and PD with the three variables just mentioned follow the same direction and roughly the same intensity. We also mention the positive correlations between the three variables (PSC, PDC, and SAP) whose r ranged from 0.19 to 0.33 (*p* < 0.001). Finally, we note that there was no significant correlation between OLMHPs, or their subscales, and age or numbers of online semesters.

## 5. Discussion

The aim of this study was to validate a scale that could assess the MHPs of university students, especially in the intensive use of online learning. This scale is short and can be easily applied in research where many variables are measured, so as not to overload the total number of items administered to participants. The measurement can indicate a higher or lower level of mental health impairment in the context of online learning. The validation process started by generating a set of 18 items to measure the OLMHPs construct. From this item pool, nine statements were removed after the expert evaluation. EFA of the remaining items revealed two distinct factors. Then, in CFA three different types of structural models were tested, a one-factor, a two-first-order correlated factor, and a second-order factor, the last of these being retained further in the analysis. The coefficients of the representative GOF indices for measuring incremental, parsimonious, and absolute fits had values above the recommended thresholds, indicating good construct validity of the scale. Also, the multiple examination of the scale showed high reliability, and good convergent and discriminant validity, with the corresponding coefficients having the accepted values.

The OLMHPs scale contains two subscales assessing two kinds of MHPs in online learning. The first of these, named *online academic difficulties*, includes indicators that describe the difficulties students experience in online learning, such as poor learning performance, difficulty concentrating, low motivation, tiredness, and feelings of social isolation. These mental issues have been mentioned in previous research regarding online context, e.g., poor performance (Liu et al., 2022), low motivation (Soria et al., 2020), fatigue (Ishimaru et al., 2021; Maloney et al., 2023), social isolation (Bączek et al., 2021), change in activity habits (De Haas et al., 2020), etc. The second subscale, named *psychological distress*, contains indicators measuring anxiety, stress, and feeling insecure. Of the three components of psychological distress, anxiety and stress appear frequently in previous research (e.g., Daumiller et al., 2023; Almeda et al., 2022; Xu & Wang, 2023).

As expected, our findings revealed a high positive correlation between OAD and PD. Numerous studies show negative associations between variables measuring psychological distress and well-being (positive affect, life satisfaction, etc.) in university students (e.g., Kraiss et al., 2023; Nogueira & Sequeira, 2024; Yotsidi et al., 2023). Since the OAD indicators can be considered as belonging to the broad sphere of well-being, through our theoretical model we anticipated this correlation. Considering the negative wording of the OAD subscale items, we consider that our results support the findings of previous research. As we showed in the theoretical background, in several studies PD has been conceptualized either separately, as a construct stand-alone, or as a dimension (sub-construct) of mental health. The OAD dimension can also be conceptualized as a sub-construct of mental health, from the perspective of (lack of) well-being. These two dimensions were put together in a unified model of mental health, a perspective that relates well-being and distress as two distinct, but correlated mental health dimensions. Moreover, the results also lead to the idea that these two constructs are subordinated not only conceptually, but also empirically, to a higher-order construct, respectively, mental health. In conclusion, there was statistical evidence and theoretical reasons to accept two conceptual levels of the OLMHPs construct. Our findings are consistent with the results of other research, which has highlighted a hierarchical factor model of mental health. For instance, Veit and Ware (1983) described a second-order factor model, with two first-order correlated factors, Psychological Distress and Psychological Well-being, and one factor at a higher level, namely Mental Health. Also, Massé et al. (1998) presented a second-order factor model of mental health having the same sub-constructs as the model mentioned above.

The correlations between OLMHPs and other variables related to online learning (PSC, PDC, and SAP) were moderate and negative, confirming our assumptions. These associations show that students who perceive themselves as having higher levels of digital competence, social competence, and academic performance tend to have lower levels of MHPs, whether we refer to the global score of the entire OLMHPs scale or to the scores of either of the two subscales. The practical implications of these results are obvious. Among these, improving learners’ digital skills through various courses (e.g., ICT skills courses), as well as compensating for their social competence deficits in various ways (e.g., more frequent positive feedback, higher level of social presence of the teacher) could be relatively easily put into practice. The data of this study do not support causal relationships, but future research could explore the determinant role of these variables and other ones that might be involved in the occurrence of mental problems in online learning.

Finally, we discuss two aspects regarding the reporting of this scale to others who assess similar MHPs. First, the question can be raised about how valid were the measurements of complex constructs, such as anxiety or perceived stress using a single-item scale. On that point, we considered the population that participated in this study to fall into the category of highly educated people. We can argue that anxiety and stress are concepts widely known, because they were passed from the academic language to the common language, and a person with even an average education is familiar with them. Research participants not only understand what these concepts refer to, but in our opinion, they can self-assess them, just as they can about academic performance, learning satisfaction, or social skills. Thus, we consider the single-item measure of anxiety and stress in the present study to be a valid assessment, relying also on the valid results of other research in which these constructs were measured in the same way (e.g., Young et al., 2007; Eddy et al., 2019). There are arguments, in general, from some authors regarding the validity of using single-item measures, as we have shown in Section 3.2. (e.g., Bowling, 2005; Allen et al., 2022). We conclude that the form of the scale (type of items, measured dimensions, length) is suitable for measuring MHPs which can occur in online learning. The second aspect is the contextualization of the PD subscale, the construction of its items presenting a notable difference in relation to traditional instruments that measure anxiety or stress. In general, the scales used to assess anxiety, for example, contain a time indication in the instructions addressed to the respondents, usually varying between a week and a month, and do not refer to a certain aspect of human activity or a specific area of existence. In our research, the anxiety item for instance was placed in the context of online learning, thus measuring a specific or context-related anxiety, without any indication of the time. In a similar manner, anxiety towards mathematics was assessed by a single-item measure, the results obtained being comparable to those of a multi-item scale, having the same purpose (Núñez-Peña et al., 2014). The other items of the scale, regarding stress, motivation, concentration, etc., were also contextualized. In this way, the respondents were oriented towards the relationship between these indicators of mental health and online learning, in order to distinguish anxiety, stress, and other aspects measured in intensive virtual courses from their manifestations in other human contexts or in general.

## 6. Conclusions

The results of this research showed that the OLMHPs have good psychometric characteristics. Although the scale is short, its composition includes two subscales with high reliability coefficients. These features make it easy to apply without affecting the variety of data that can be obtained. There are no reversed items, the instructions for respondents are relatively simple, and the response form, i.e., the five-point Likert scale, is the same for all items.

The scale can be used to measure MHPs that may arise if online learning is used intensively, as happened during the pandemic or in the immediate aftermath, but not only for such situations. In our opinion, online learning will play, even in the absence of emergency situations, an increasing role in university teaching activities in the years to come. There is a consensus among scholars that online learning has multiple benefits, but as we have shown in this study, MHPs can also arise. The scale that we developed and validated can be very useful in measuring these mental issues. For example, the results of a study carried out before the pandemic showed that MHPs in adolescents, were associated with increasing screen time (Bor et al., 2014). In other words, the scale is not only intended to measure MHPs in ERL, which is a temporary form for the delivery of educational content, but also in online learning conducted in common life circumstances. We have chosen a period of data collection outside the lockdown in which students attended both online and face-to-face courses, allowing them to compare the two modes of content delivery. Undoubtedly, ERL was a good solution to continue academic activities during the crisis period, but intensive use of online courses, sometimes when there is the possibility of face-to-face courses, may arise several topics for reflection and research. Among these, the emergence of some mental health issues, the need to evaluate the satisfaction of the learners with the online system, as well as the identification of ways of conducting online didactic activities that limit the negative effects on mental health and increase satisfaction deserve greater attention in future research. Therefore, one way to reduce the risk of MHPs in online learning is to create and support attractive courses by teachers, with tasks that involve students, by using methods that allow dialog and participation in joint activities, such as debate, conversation, or teamwork.

The OLMHPs scale is not created for diagnosis, but rather for assessing early signs of MHPs. Further, complex scales can be applied, with an appropriate clinical diagnostic function. Also, the scale can be useful in research that aims to investigate MHPs in the context of online learning in relation to other variables of interest, such as social presence, academic self-efficacy and self-regulation, learners’ satisfaction, interest, and so on.

The study has several limitations, mainly related to the sample used. First of all, the most important limitation is the gender disproportion (86.7% female). The students who answered the questionnaire mostly came from faculties where the majority are represented by women. It is possible that the gender variable may have some impact on the research results, as there are differences between men and women in some components of distress measured during the pandemic period. For example, the results of a year-long study show the same level of depressive symptoms in males and females, but higher levels of stress and anxious symptoms in females (Arcand et al., 2023). Secondly, the OLMHP was validated on a Romanian student sample, coming exclusively from a single university. Online teaching activities were managed, both during and after the pandemic, probably in different ways, depending on various contextual factors. It is possible, for instance, that the characteristics of our university (e.g., how the activities are planned, the level of teacher training, the type of online learning platforms used, etc.) had an impact on the results of our research. Thus, we cannot know to what extent these results are also valid for other universities, but the data obtained in the process of validating the scale make us optimistic in this regard. Also, the scale was validated for university online learning, not for other levels of education, such as high school, where the online system was also widely used during the pandemic. Further research is needed with a more balanced sample composition, in terms of gender, educational context, academic specialties, and level of education (middle, secondary, and tertiary).

## Figures and Tables

**Table 1:** Sample characteristics (N = 398).

Variable		
Gender	Female	345 (86.7%)
	Male	53 (13.3%)
Age (mean, SD)		22.3 (3.50)
Number of semesters online	1	6 (1.5%)
	2	11 (2.8%)
	3	63 (15.8%)
	4	318 (79.9%)
Faculty	Letters	155 (38.9%)
	Computer Science	34 (8.5%)
	Theology	14 (3.5%)
	Mathematics	20 (5.0%)
	Law	51 (12.8%)
	Psychology	32 (8.0%)
	Philosophy	66 (16.6%)
	Economics	16 (4.0%)
	Biology	10 (2.5%)

**Table 2:** Descriptive statistics (initial form of the scale).

Item	Min	Max	Mean	SD	Skewness	Kurtosis
Item 1	1	5	2.58	1.35	0.37	−1.08
Item 2	1	5	2.30	1.34	0.70	−0.75
Item 3	1	5	2.22	1.33	0.82	−0.57
Item 4	1	5	2.55	1.40	0.41	−1.13
Item 5	1	5	3.06	1.44	−0.05	−1.32
Item 6	1	5	2.80	1.45	0.22	−1.26
Item 7	1	5	3.12	1.47	−0.12	−1.35
Item 8	1	5	2.78	1.42	0.21	−1.25
Item 9	1	5	2.79	1.48	0.15	−1.37

**Table 3:** OLMHPs EFA results.

Item	λ		h^2^	v	α	ω
	F_1_	F_2_				
Item 5. I have difficulty concentrating during online classes	**0.779**	0.280	0.587	32.76	0.88	0.88
Item 8. In online learning, my work performance is poor	**0.730**	0.292	0.545			
Item 6. I do not feel motivated to get involved in online classes	**0.712**	0.312	0.537			
Item 7. I get tired quickly when taking online classes	**0.671**	0.377	0.550			
Item 9. When learning takes place online, I experience the feeling of social isolation	**0.658**	0.399	0.541			
Item 3. Taking online classes causes me stress	0.307	**0.844**	0.661	28.80	0.84	0.85
Item 2. Taking online classes gives me anxiety	0.318	**0.792**	0.645			
Item 1. In online learning, the virtual environment makes me feel insecure	0.389	**0.644**	0.547			
Item 4. I am more worried than usual during online classes *	0.265	**0.525**	0.361			

EFA extraction method: PAF; λ—factor loadings; h^2^—communalities; v—% of variance explained; α—Cronbach’s alpha; ω—McDonald’s omega; * item eliminated before CFA.

**Table 4:** OLMHPs—standardized factor loadings, reliability, and convergent validity.

OLMHPs Items	Subscale	λ	α	ω	AVE
Item 5	Online academic difficulties	0.81	0.88	0.88	0.61
Item 8		0.78			
Item 6		0.78			
Item 7		0.77			
Item 9		0.77			
Item 3	Psychological distress	0.85	0.87	0.87	0.69
Item 2		0.87			
Item 1		0.78			

λ—Standardized factor loadings; α—Cronbach’s alpha; ω—McDonald’s omega; AVE—average variance extracted.

**Table 5:** Correlations of OLMHPs scale and subscales with other measures.

	1	2	3	4	5	6	7
1. Perceived social competence	1						
2. Perceived digital competence	0.33 **	1					
3. Subjective academic performance	0.27 **	0.32 **	1				
4. Online learning satisfaction	0.20 **	0.27 **	0.19 **	1			
5. Online academic difficulties	−0.19 **	−0.22 **	−0.23 **	−0.58 **	1		
6. Psychological distress	−0.12 *	−0.27 **	−0.20 **	−0.57 **	0.66 **	1	
7. OLMHPs	−0.18 **	−0.27 **	−0.24 **	−0.62 **	0.95 **	0.86 **	1

* *p* < 0.01, ** *p* < 0.001.

## Data Availability

Data are available from the authors on request.

## Appendix A

Below are eight statements about online learning. Using the 1–5 scale, please indicate your agreement with each statement. There are no right or wrong answers.

1 = strongly disagree, 2 = disagree, 3 = neither agree nor disagree, 4 = agree, 5 = strongly agree

In online learning, the virtual environment makes me feel insecure.
Taking online classes gives me anxiety.
Taking online classes causes me stress.
I have difficulty concentrating during online classes.
In online learning, my work performance is poor.
I do not feel motivated to get involved in online classes.
I get tired quickly when taking online classes.
When learning takes place online, I experience the feeling of social isolation.

## References

[B1-behavsci-15-00026] Ahorsu D. K., Lin C. Y., Imani V., Saffari M., Griffiths M. D., Pakpour A. H. (2020). The fear of COVID-19 scale: Development and initial validation. International Journal of Mental Health and Addiction.

[B2-behavsci-15-00026] Aisha N., Ratra A. (2022). Online education amid COVID-19 pandemic and its opportunities, challenges and psychological impacts among students and teachers: A systematic review. Asian Association of Open Universities Journal.

[B3-behavsci-15-00026] Alam M. D., Lu J., Ni L., Hu S., Xu Y. (2021). Psychological outcomes and associated factors among the international students living in China during the COVID-19 pandemic. Frontiers in Psychiatry.

[B4-behavsci-15-00026] Alexander M. W., Truell A. D., Zhao J. J. (2012). Expected advantages and disadvantages of online learning: Perceptions from college students who have not taken online courses. Issues in Information Systems.

[B5-behavsci-15-00026] Allen M. S., Iliescu D., Greiff S. (2022). Single item measures in psychological science: A call to action. European Journal of Psychological Assessment.

[B6-behavsci-15-00026] Almeda N., Díaz-Milanés D., Guiterrez-Colosia M. R., García-Alonso C. R. (2022). A systematic review of the international evolution of online mental health strategies and recommendations during the COVID-19 pandemic. BMC Psychiatry.

[B8-behavsci-15-00026] American Psychiatric Association (2013). Diagnostic and statistical manual of mental disorders.

[B7-behavsci-15-00026] American Psychological Association (APA) (2018). APA dictionary of psychology.

[B9-behavsci-15-00026] Antaramian S. (2015). Assessing psychological symptoms and well-being: Application of a dual-factor mental health model to understand college student performance. Journal of Psychoeducational Assessment.

[B10-behavsci-15-00026] Arcand M., Bilodeau-Houle A., Juster R.-P., Marin M.-F. (2023). Sex and gender role diferences on stress, depression, and anxiety symptoms in response to the COVID-19 pandemic over time. Frontiers in Psychology.

[B12-behavsci-15-00026] Bates R., Khasawneh S. (2007). Self-efficacy and college students’ perceptions and use of online learning systems. Computers in Human Behavior.

[B13-behavsci-15-00026] Bayrak F., Tibi M. H., Altun A. (2020). Development of Online Course satisfaction Scale. Turkish Online Journal of Distance Education.

[B11-behavsci-15-00026] Bączek M., Zagańczyk-Bączek M., Szpringer M., Jaroszyński A., Wożakowska-Kapłon B. (2021). Students’ perception of online learning during the COVID-19 pandemic: A survey study of Polish medical students. Medicine.

[B14-behavsci-15-00026] Beserra V., Nussbaum M., Navarrete M., Garrido N. (2022). Online physically active academic lessons in COVID-19 times: A pilot study. Teaching and Teacher Education.

[B15-behavsci-15-00026] Bor W., Dean A. J., Najman J., Hayatbakhsh R. (2014). Are child and adolescent mental health problems increasing in the 21st century? A systematic review. Australian and New Zealand Journal of Psychiatry.

[B16-behavsci-15-00026] Bowling A. (2005). Just one question: If one question works, why ask several?. Journal of Epidemiology and Community Health.

[B17-behavsci-15-00026] Brown T. A. (2015). Confirmatory factor analysis for applied research.

[B18-behavsci-15-00026] Buizza C., Bazzoli L., Ghilardi A. (2022). Changes in college students mental health and lifestyle during the COVID-19 pandemic: A systematic review of longitudinal studies. Adolescent Research Review.

[B19-behavsci-15-00026] Burnette J. L., Knouse L. E., Vavra D. T., O’Boyle E., Brooks M. A. (2020). Growth Mindsets and psychological distress: A meta-analysis. Clinical Psychology Review.

[B20-behavsci-15-00026] Byrne B. M. (2016). Structural equation modelling with AMOS: Basic concepts, applications, and programming.

[B21-behavsci-15-00026] Centers for Disease Control and Prevention (2024). About mental health?.

[B22-behavsci-15-00026] Chaffee B. W., Cheng J., Couch E. T., Halpern-Felsher B. (2024). Engagement, Mental Health, and Substance Use Under In-Person or Remote School Instruction During the COVID-19 Pandemic. Journal of School Health.

[B23-behavsci-15-00026] Cheung G. W., Cooper-Thomas H. D., Lau R. S., Wang L. C. (2024). Reporting reliability, convergent and discriminant validity with structural equation modeling: A review and best-practice recommendations. Asia Pacific Journal of Management.

[B24-behavsci-15-00026] Cloninger C. R. (2006). The science of well-being: An integrated approach to mental health and its disorders. World Psychiatry.

[B25-behavsci-15-00026] Cofini V., Perilli E., Moretti A., Bianchini V., Perazzini M., Muselli M., Lanzi S., Tobia L., Fabiani L., Necozione S. (2022). E-Learning Satisfaction, Stress, Quality of Life, and Coping: A Cross-Sectional Study in Italian University Students a Year after the COVID-19 Pandemic Began. International Journal of Environmental Research and Public Health.

[B26-behavsci-15-00026] Collie R. J. (2020). The development of social and emotional competence at school: An integrated model. International Journal of Behavioral Development.

[B27-behavsci-15-00026] Curelaru M., Curelaru V., Cristea M. (2022). Students’ perceptions of online learning during COVID-19 pandemic: A qualitative approach. Sustainability.

[B28-behavsci-15-00026] Daumiller M., Rinas R., Schoon I., Lüftenegger M. (2023). How did COVID-19 affect education and what can be learned moving forward? A systematic meta-review of systematic reviews and meta-analyses. Zeitschrift für Psychologie.

[B29-behavsci-15-00026] De Haas M., Faber R., Hamersma M. (2020). How COVID-19 and the Dutch ‘intelligent lockdown’change activities, work and travel behaviour: Evidence from longitudinal data in the Netherlands. Transportation Research Interdisciplinary Perspectives.

[B30-behavsci-15-00026] de la Fuente J., Sander P., Kauffman D. F., Yilmaz Soylu M. (2020). Differential effects of self-vs. external-regulation on learning approaches, academic achievement, and satisfaction in undergraduate students. Frontiers in Psychology.

[B31-behavsci-15-00026] Diotaiuti P., Valente G., Mancone S., Bellizzi F. (2021). A Mediating Model of Emotional Balance and Procrastination on Academic Performance. Frontiers in Psychology.

[B32-behavsci-15-00026] Drapeau A., Marchand A., Beaulieu-Prévost D., L’Abate P. L. (2012). Epidemiology of Psychological Distress. Mental illnesses-understanding, prediction and control.

[B33-behavsci-15-00026] Dumford A. D., Miller A. L. (2018). Online learning in higher education: Exploring advantages and disadvantages for engagement. Journal of Computing in Higher Education.

[B34-behavsci-15-00026] Eddy C. L., Herman K. C., Reinke W. M. (2019). Single-item teacher stress and coping measures: Concurrent and predictive validity and sensitivity to change. Journal of School Psychology.

[B35-behavsci-15-00026] Eklund K., Dowdy E., Jones C., Furlong M. (2011). Applicability of the dual-factor model of mental health for college students. Journal of College Student Psychotherapy.

[B36-behavsci-15-00026] Eleftheriades R., Fiala C., Pasic M. D. (2020). The challenges and mental health issues of academic trainees. F1000Research.

[B37-behavsci-15-00026] Elshami W., Taha M. H., Abuzaid M., Saravanan C., Al Kawas S., Abdalla M. E. (2021). Satisfaction with online learning in the new normal: Perspective of students and faculty at medical and health sciences colleges. Medical Education Online.

[B38-behavsci-15-00026] European Commission (2019). Key competences for lifelong learning.

[B39-behavsci-15-00026] Fisher G. G., Matthews R. A., Gibbons A. M. (2016). Developing and investigating the use of single-item measures in organizational research. Journal of Occupational Health Psychology.

[B40-behavsci-15-00026] Fornell C., Larcker D. F. (1981). Evaluating structural equation models with unobservable variables and measurement errors. Journal of Marketing Research.

[B41-behavsci-15-00026] Garcia A., Powell G. B., Arnold D., Ibarra L., Pietrucha M., Thorson M. K., Verhelle A., Wade N. B., Webb S. (2021). Learned helplessness and mental health issues related to distance learning due to COVID-19. Extended abstracts of the 2021 chi conference on human factors in computing systems.

[B42-behavsci-15-00026] Greenspoon P. J., Saklofske D. H. (2001). Toward an integration of subjective well-being and psychopathology. Social Indicators Research.

[B43-behavsci-15-00026] Gunzler D. D., Perzynski A. T., Carle A. C. (2021). Structural equation modeling for health and medicine.

[B44-behavsci-15-00026] Hair J. F., Black W. C., Babin B. J., Anderson R. E. (2019). Multivariate data analysis.

[B45-behavsci-15-00026] Hasan N., Bao Y. (2020). Impact of “e-Learning crack-up” perception on psychological distress among college students during COVID-19 pandemic: A mediating role of “fear of academic year loss”. Children and Youth Services Review.

[B46-behavsci-15-00026] Hayes A. F., Coutts J. J. (2020). Use Omega Rather than Cronbach’s Alpha for Estimating Reliability. But…. Communication Methods and Measures.

[B47-behavsci-15-00026] Henseler J., Ringle C. M., Sarstedt M. (2015). A New Criterion for Assessing Discriminant Validity in Variance-Based Structural Equation Modeling. Journal of the Academy of Marketing Science.

[B48-behavsci-15-00026] Hooper D., Coughlan J., Mullen M. (2008). Structural Equation Modelling: Guidelines for Determining Model Fit. Electronic. Journal of Business Research Methods.

[B49-behavsci-15-00026] Hu L.-T., Bentler P. M. (1999). Cutoff criteria for fit indexes in covariance structure analysis: Conventional criteria versus new alternatives. Structural Equation Modeling.

[B50-behavsci-15-00026] Huang C., Tu Y., He T., Han Z., Wu X. (2024). Longitudinal exploration of online learning burnout: The role of social support and cognitive engagement. European Journal of Psychology of Education.

[B51-behavsci-15-00026] Huppert F. A. (2014). The state of wellbeing science. Concepts, measures, interventions, and policies.

[B52-behavsci-15-00026] Huppert F. A., So T. T. C. (2013). Flourishing Across Europe: Application of a New Conceptual Framework for Defining Well-Being. Social Indicators Research.

[B53-behavsci-15-00026] Iasiello M., Agteren J. V., Muir-Cochrane E. (2020). Mental Health and/or Mental Illness: A Scoping Review of the Evidence and Implications of the Dual-Continua Model of Mental Health. Evidence Base.

[B54-behavsci-15-00026] Institutul National de Sanatate Publica (n.d.). Despre sănătatea mintală a adulților tineri.

[B55-behavsci-15-00026] Ishimaru D., Adachi H., Nagahara H., Shirai S., Takemura H., Takemura N., Mehrasa A., Higashino T., Yagi Y., Ikeda M. (2021). Characteristics of Adaptation in Undergraduate University Students Suddenly Exposed to Fully Online Education During the COVID-19 Pandemic. Frontiers in Psychiatry.

[B57-behavsci-15-00026] Keyes C. L. M. (2005). Mental illness and/or mental health? Investigating axioms of the complete state model of health. Journal of Consulting and Clinical Psychology.

[B56-behavsci-15-00026] Keyes C. L. M. (2014). Mental Health as a Complete State: How the Salutogenic Perspective Completes the Picture. Bridging occupational, organizational and public health.

[B58-behavsci-15-00026] Kim H. Y. (2013). Statistical notes for clinical researchers: Assessing normal distribution (2) using skewness and kurtosis. Restorative Dentistry & Endodontics.

[B59-behavsci-15-00026] Kim S., Sturman E., Kim E. S., Strang K. D. (2015). Structural Equation Modeling: Principles, Processes, and Practices. The palgrave handbook of research design in business and management.

[B60-behavsci-15-00026] Knapstad M., Sivertsen B., Knudsen A. K., Smith O. R. F., Aarø L. E., Lønning K. J., Skogen J. C. (2021). Trends in self-reported psychological distress among college and university students from 2010 to 2018. Psychological Medicine.

[B61-behavsci-15-00026] Kraiss J. T., Kohlhoff M., ten Klooster P. M. (2023). Disentangling between- and within-person associations of psychological distress and mental well-being: An experience sampling study examining the dual continua model of mental health among university students. Current Psychology.

[B62-behavsci-15-00026] Kumar H., Shaheen A., Rasool I., Shafi M. (2016). Psychological Distress and Life Satisfaction among University Students. Journal of Psychology and Clinical Psychiatry.

[B63-behavsci-15-00026] Lee J., Solomon M., Stead T., Kwon B., Ganti L. (2021). Impact of COVID-19 on the mental health of US college students. BMC Psychology.

[B64-behavsci-15-00026] Leung S. O., Xu M. L. (2013). Single-Item Measures for Subjective Academic Performance, Self-Esteem, and Socioeconomic Status. Journal of Social Service Research.

[B65-behavsci-15-00026] Liu X., Gong Z., Miao K., Yang P., Liu H., Feng Z., Chen Z. (2022). Attitude and Performance for Online Learning during COVID-19 Pandemic: A Meta-Analytic Evidence. International Journal of Environmental Research and Public Health.

[B66-behavsci-15-00026] Lucas M., Vicente P. N. (2023). A double-edged sword: Teachers’ perceptions of the benefits and challenges of online teaching and learning in higher education. Education and Information Technologies.

[B67-behavsci-15-00026] Maloney S., Axelsen M., Stone C., Galligan L., Redmond P., Brown A., Turner J., Lawrence J. (2023). Defining and exploring online engagement fatigue in a university context. Computers and Education Open.

[B68-behavsci-15-00026] Massé R., Poulin C., Dassa C., Lambert J., Bélair S., Battaglini A. (1998). The structure of mental health: Higher-order confirmatory factor analyses of psychological distress and well-being measures. Social Indicators Research.

[B69-behavsci-15-00026] McDonald R. P. (1999). Test theory: A unified treatment.

[B70-behavsci-15-00026] Mirowsky J., Ross C. E. (2003). Social causes of psychological distress.

[B71-behavsci-15-00026] Mishra L., Gupta T., Shree A. (2020). Online teaching-learning in higher education during lockdown period of COVID-19 pandemic. International Journal of Educational Research Open.

[B72-behavsci-15-00026] Naseer S., Perveen H. Z. (2023). Perspective chapter: Advantages and disadvantages of online learning courses. Massive open online courses-current practice and future trends.

[B73-behavsci-15-00026] National Institute of Mental Health (2024). Mental illness.

[B74-behavsci-15-00026] Naylor R. (2020). Key factors influencing psychological distress in university students: The effects of tertiary entrance scores. Studies in Higher Education.

[B75-behavsci-15-00026] NHS England (2022). Mental health of children and young people in england 2022—Wave 3 follow up to the 2017 survey.

[B77-behavsci-15-00026] Núñez-Peña M. I., Guilera G., Suárez-Pellicioni M. (2014). The Single-Item Math Anxiety Scale: An Alternative Way of Measuring Mathematical Anxiety. Journal of Psychoeducational Assessment.

[B76-behavsci-15-00026] Nogueira M. J. C., Sequeira C. A. (2024). Positive and Negative Correlates of PsychologicalWell-Being and Distress in College Students Mental Health: A Correlational Study. Healthcare.

[B78-behavsci-15-00026] Nunnally J. C., Bernstein I. H. (1994). Psychometric theory.

[B79-behavsci-15-00026] Pai N., Vella S.-L. (2021). COVID-19 and loneliness: A rapid systematic review. Australian & New Zealand Journal of Psychiatry.

[B80-behavsci-15-00026] Peitz D., Kersjes C., Christina, Thom J., Heike Hoelling H., Mauz E. (2021). Indicators for Public Mental Health: A Scoping Review. Frontiers Public Health.

[B81-behavsci-15-00026] Pengpid S., Peltzer K. (2020). Psychological distress and its associated factors among school-going adolescents in Tanzania. Psychological Studies.

[B82-behavsci-15-00026] Robins R. W., Hendin H. M., Trzesniewski K. H. (2001). Measuring Global Self-Esteem: Construct Validation of a Single-Item Measure and the Rosenberg Self-Esteem Scale. Personality and Social Psychology Bulletin.

[B83-behavsci-15-00026] Rubin K. H., Rose-Krasnor L., Van Hasselt V. B., Hersen M. (1992). Interpersonal problem solving and social competence in children. Handbook of social development: A lifespan perspective.

[B84-behavsci-15-00026] Schmits E., Dekeyser S., Klein O., Luminet O., Yzerbyt V., Glowacz F. (2021). Psychological Distress among Students in Higher Education: One Year after the Beginning of the COVID-19 Pandemic. International Journal of Environmental Research and Public Health.

[B85-behavsci-15-00026] Schramme T. (2010). Can we define mental disorder by using the criterion of mental dysfunction?. Theoretical Medicine and Bioethics.

[B86-behavsci-15-00026] Schreiber J. B., Nora A., Stage F. K., Barlow E. A., King J. (2006). Reporting Structural Equation Modeling and Confirmatory Factor Analysis Results: A Review. The Journal of Educational Research.

[B87-behavsci-15-00026] Smith D., McLellan R. (2023). Mental health problems in first-generation university students: A scoping review. Review of Education.

[B88-behavsci-15-00026] Song L., Singleton E. S., Hill J. R., Koh M. H. (2004). Improving online learning: Student perceptions of useful and challenging characteristics. The Internet and Higher Education.

[B89-behavsci-15-00026] Soria K. M., Chirikov I., Jones-White D. (2020). The obstacles to remote learning for undergraduate, graduate, and professional students.

[B90-behavsci-15-00026] Steiger J. H. (2007). Understanding the Limitations of Global Fit Assessment in Structural Equation Modeling. Personality and Individual Differences.

[B91-behavsci-15-00026] Tannenbaum C., Lexchin J., Tamblyn R., Romans S. (2009). Indicators for measuring mental health: Towards better surveillance. Healthcare Policy.

[B95-behavsci-15-00026] Veit C. T., Ware J. E. (1983). The structure of psychological distress and well-being in general populations. Journal of Consulting and Clinical Psychology.

[B92-behavsci-15-00026] Wang S., Chen L., Ran H., Che Y., Fang D., Sun H., Peng J., Liang X., Xiao Y. (2022). Depression and anxiety among children and adolescents pre and post COVID-19: A comparative meta-analysis. Frontiers in Psychiatry.

[B93-behavsci-15-00026] Wang X., Tan S. C., Li L. (2020). Technostress in university students’ technology-enhanced learning: An investigation from multidimensional person-environment misfit. Computers in Human Behavior.

[B94-behavsci-15-00026] Wathelet M., Horn M., Creupelandt C., Fovet T., Baubet T., Habran E., Martignène N., Vaiva G., D’hondt F. (2022). Mental health symptoms of university students 15 months after the onset of the COVID-19 pandemic in France. JAMA Network Open.

[B96-behavsci-15-00026] World Health Organization (2022). World mental health report.

[B97-behavsci-15-00026] World Health Organizațion (2024). Mental health of adolescents.

[B98-behavsci-15-00026] Xiao R., Zhang C., Lai Q., Hou Y., Zhang X. (2021). Applicability of the Dual-Factor Model of Mental Health in the Mental Health Screening of Chinese College Students. Frontiers in Psychology.

[B99-behavsci-15-00026] Xu T., Wang H. (2023). High prevalence of anxiety, depression, and stress among remote learning students during the COVID-19 pandemic: Evidence from a meta-analysis. Frontiers in Psychology.

[B100-behavsci-15-00026] Yotsidi V., Nikolatou E.-K., Kourkoutas E., Kougioumtzis G. A. (2023). Mental distress and well-being of university students amid COVID-19 pandemic: Findings from an online integrative intervention for psychology trainees. Frontiers in Psychology.

[B101-behavsci-15-00026] Young Q. R., Ignaszewski A., Fofonoff D., Kaan A. (2007). Brief screen to identify 5 of the most common forms of psychosocial distress in cardiac patients: Validation of the screening tool for psychological distress. Journal of Cardiovascular Nursing.

[B102-behavsci-15-00026] Zhao M. Y., Tay L. (2023). From ill-being to well-being: Bipolar or bivariate?. The Journal of Positive Psychology.

[B103-behavsci-15-00026] Zhou J., Wang Y., Bu T., Zhang S., Chu H., Li J., He J., Zhang Y., Liu X., Qiao Z., Yang X., Yang Y. (2021). Psychological impact of COVID-19 epidemic on adolescents: A large sample study in China. Frontiers in Psychiatry.

[B104-behavsci-15-00026] Zhu Y., Jha S. C., Shutta K. H., Huang T., Balasubramanian R., Clish C. B., Hankinson S. E., Kubzansky L. D. (2022). Psychological distress and metabolomic markers: A systematic review of posttraumatic stress disorder, anxiety, and subclinical distress. Neuroscience & Biobehavioral Reviews.

